# Aryl hydrocarbon receptor activation ameliorates experimental colitis by modulating the tolerogenic dendritic and regulatory T cell formation

**DOI:** 10.1186/s13578-022-00780-z

**Published:** 2022-04-23

**Authors:** Xiufang Cui, Ziping Ye, Di Wang, Yan Yang, ChunHua Jiao, Jingjing Ma, Nana Tang, Hongjie Zhang

**Affiliations:** 1 Department of Gastroenterology, Nanjing First Hospital, Nanjing Medical University, Nanjing, Jiangsu Province China; 2 Department of Gastroenterology, First Affiliated Hospital of Nanjing Medical University, 300# Guangzhou Road, Nanjing, 210029 Jiangsu People’s Republic of China; 3 Department of Gastroenterology, Sir Run Run Hospital, Nanjing Medical University, Nanjing, 211100 Jiangsu People’s Republic of China

**Keywords:** Aryl hydrocarbon receptor, Crohn’s disease, Dendritic cells, Intestinal immune tolerance, Treg cells

## Abstract

**Background:**

Intestinal immune dysfunction is involved in the onset of Crohn’s disease (CD). Dendritic cells (DCs), antigen-presenting cells, play a key role in the maintenance of intestinal immune homeostasis. The aryl hydrocarbon receptor (AhR) is a ligand-dependent transcription factor widely expressed in various immune cells, including DCs. Although AhR plays an important role in immune tolerance, its role in the DCs is unclear. The purpose of this study was to investigate whether the activation of AhR can induce tolerogenic DCs (tolDCs) and the differentiation of regulatory T (Treg) cells, as well as ameliorate experimental colitis.

**Results:**

AhR activation in the DCs resulted in a lower expression of surface markers such as CD80, CD83, CD86, and pro-inflammatory cytokine production, and higher anti-inflammatory production (IL-1β, IL-23, and IL-12) compared to the control DCs. The surface dendrites in DCs were significantly reduced following AhR activation by 6-formylindolo [3,2-b]carbazole (FICZ). Such DCs with FICZ-mediated activation of AhR, namely tolDCs, promoted Treg cell differentiation. Adoptive transfer of tolDCs to a TNBS-induced colitis mouse model significantly alleviated the severity of inflammation by improving the colon length and decreasing the disease activity index (DAI) and histopathological score. Moreover, the transferred tolDCs decreased the frequency of Th17 cells and increased the frequency of Treg cells in the spleen and mesenteric lymph nodes (MLNs) in murine colitis models.

**Conclusions:**

Activation of AhR in the DCs could induce tolDCs, and the transplantation of tolDCs may help in relieving intestinal inflammation and maintaining the Th17/Treg differentiation balance. Thus, our data suggest that AhR may be a potential therapeutic target for CD.

## Background

Crohn's disease (CD) is a chronic non-specific intestinal inflammatory disease. Although its etiology and pathogenesis remain unclear, a large amount of evidence indicates that the pathological mechanisms of CD are driven by an exaggerated immune imbalance [1]. T helper 17 (Th17) cells and regulatory T (Treg) cells are two vital lymphocyte subsets with opposing functions. While the Th17 cells have important implications in autoimmune diseases, the Treg cells control the autoimmune reactivities [2]. Mechanically, the frequency of Th17 cells was increased, while that of Treg cells was decreased in the intestinal mucosa of CD patients [3]. Previous studies have demonstrated that an imbalance of Th17 and Treg cells is associated with the development of CD [4, 5]. Thus, therapeutic regimens regulating the Th17/Treg cell balance may be used for the treatment of CD.

Dendritic cells (DCs) are the most important antigen-presenting cells acting as regulators of the T cell response involved in bridging the innate and adaptive immune responses. DCs recognize and process antigens, and trigger T cell-mediated immune responses. Further, they affect T cell differentiation and functions by upregulating the major histocompatibility complex (MHC) and costimulatory molecules such as CD80, CD83, and CD86, that play a crucial role in maintaining immune tolerance, including the Th17 and Treg cell balance [6, 7]. Tolerogenic dendritic cells (tolDCs) show decreased expression of costimulatory molecules and pro-inflammatory cytokines, and increased levels of anti-inflammatory cytokines, which in turn inhibit T cell priming and proliferation [8]. It has been previously shown that growth factors, immunosuppressants, and cytokines are involved in the induction of tolDCs tolerance [9, 10]. Some transcription factors, such as nuclear factor κB (NF-κB) and aryl hydrocarbon receptor (AhR), are also involved in the differentiation of tolDCs [11]. However, the roles of nuclear transcription factors, especially AhR, in DCs maturation and function remain largely unknown.

The role of AhR, a ligand-dependent transcription activator, in the differentiation, maturation, and function of various immune cells, has been previously investigated [11]. AhR can be activated by various endogenous and exogenous ligands and leads to a variety of immune regulatory effects. Examples of endogenous ligands that can activate AhR include 6-for mylindolo [3,2-b]carbazole (FICZ), 2-(1'H-indole-3'-carbonyl)-thiazole-4-carboxylic acid methyl ester (ITE) and exogenous ligands include 3-methylcholanthrene (3-MC) and 2,3,7,8-tetrachlorodibenzo-p-dioxin (TCDD) [11, 12]. The activated AhR is translocated into the nucleus to initiate transcription of the CYP1A1 gene [12]. Therefore, the expression levels of CYP1A may be a marker of AhR activation. AhR ligands, such as FICZ and ITE, have been shown to attenuate experimental colitis and restore the Th17 /Treg cell balance by inhibiting Th17 cell differentiation and promoting Treg cell formation [13, 14]. A previous study demonstrated that ITE could inhibit T cell-mediated immunity by acting on the DCs [15]. Another study found that FICZ could inhibit DC-primed Th17 polarization from naïve T cells [16]. These data suggest that AhR activation may affect the function of DCs and mediate inflammation associated with CD. However, the regulatory mechanisms of AhR on DCs remain largely unclear. Therefore, this study focuses on exploring the potential mechanisms underlying the AhR activation in DCs playing a crucial role in the regulation of auto-reactive effector T and Treg cells.

We hypothesized that AhR activation induces the tolDCs by downregulating costimulatory molecules such as CD80, CD83, CD86, and inflammatory cytokines that may prevent the pathogenic processes underlying colitis in mice. Here, we investigated the effects of AhR activation by FICZ on the maturation and functions of DCs in vitro and found that the FICZ-treated DCs exhibited tolerogenic properties, namely tolDCs. Furthermore, the tolDCs were transferred to a 2, 4, 6-trinitrobenzene sulfonic acid (TNBS)-induced murine model of colitis, and their impact on Th17/Treg cell balance was analyzed. These data show that the adoptive transfer of tolDCs alleviates TNBS- induced experimental colitis in mice, and this effect may be closely related to restoring the balance of Th17/Treg cell differentiation in vivo. Thus, our data suggest that AhR may be a potential therapeutic target for CD by inducing tolDCs.

## Results

### FICZ as a ligand can effectively activate AhR in dendritic cells

**Fig. 1:**
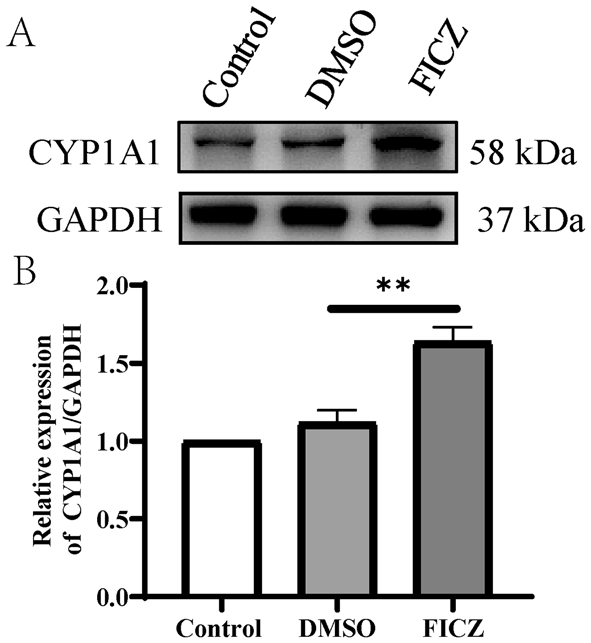
Effect of FICZ on the expression of CYP1A1 in DCs by Western blotting. BMDCs were treated with DMSO or FICZ. The levels of activated AhR in the DCs were determined by assessing CYP1A1 protein expression using Western blotting (CYP1A1 expression reflects AhR activation level). GAPDH is used as an internal control. **A** The Western Blot image of CYP1A1 of the treated DCs; **B** The relative intensity for bands of CYP1A1 normalized to that of GAPDH. Data are represented as Mean ± SD of three independent experiments. ^**^P < 0.01 versus the DMSO group

To observe the AhR activation, we analyzed CYP1A1 expression (an AhR activation marker protein) by Western blotting. CYP1A1 expression was significantly upregulated by FICZ (final concentration 100 nM)- treated DCs compared to DMSO-treated DCs (Fig. 1A, B). The results indicated that AhR could be effectively activated by its ligand FICZ in the DCs.

### Influence of FICZ-mediated AhR activation on the dendritic cell phenotype

**Fig. 2:**
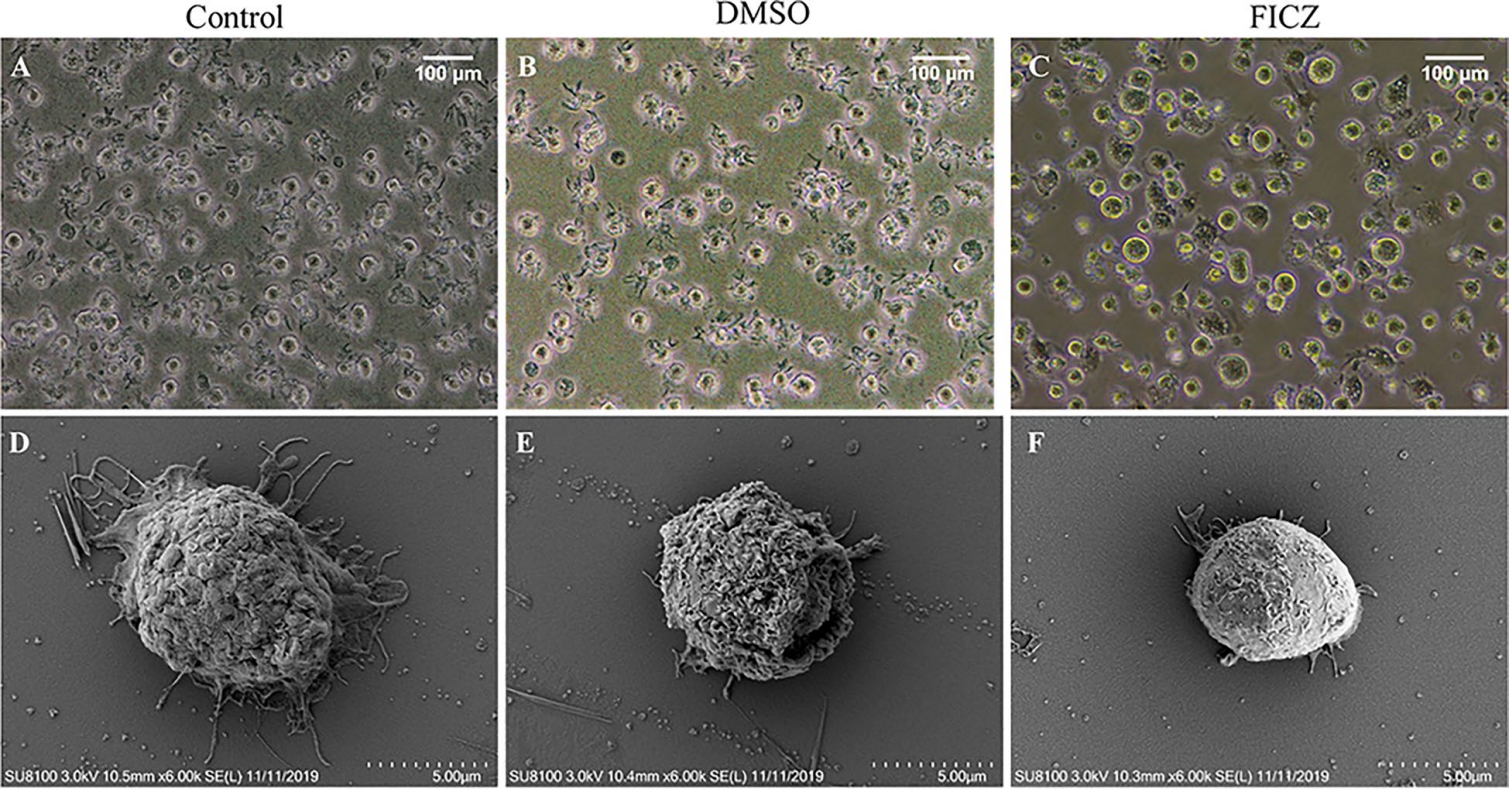
Morphological characteristics of typical and FICZ-treated DCs by light and scanning electron microscopy. BMDCs were conditioned with cytokines (GM-CSF and IL-4) and treated with DMSO (as vehicle control) or FICZ at the indicated concentration of 100 nM for 12 h, and further maturation was induced using LPS for another 24 h. **A**, **B**, **C** Light microscopic analysis of the DCs, respectively (Scale bar = 100 μm). **D**, **E**, **F** Scanning electron microscopy of Control, DMSO, and FICZ- treated DCs, respectively (Scale bar = 5 μm)

Dendritic cells contain very obvious dendrite that facilitate contact with the T cells [17]. In this study, we investigated the effects of FICZ- mediated AhR activation on the maturation of DCs in vitro. As shown in Fig. 2A, it was found that the mature DCs showed quite obvious dendrite through ordinary light microscopy. Treatment with DMSO did not change the morphology of DCs (Fig. 2B), while the DCs pretreated with 100 nM FICZ displayed small dendrites compared to the control DCs (Fig. 2C). Furthermore, scanning electron microscopy showed that massive dendrite formation on the surface of mature DCs (Fig. 2D), while the surface dendrites were significantly reduced after pretreatment with FICZ (100 nM) for 12 h. The morphology of FICZ-conditioned DCs differed from control and DMSO-treated DCs phenotypes, at least in formatting dendrite on the cell surface.

**Fig. 3:**
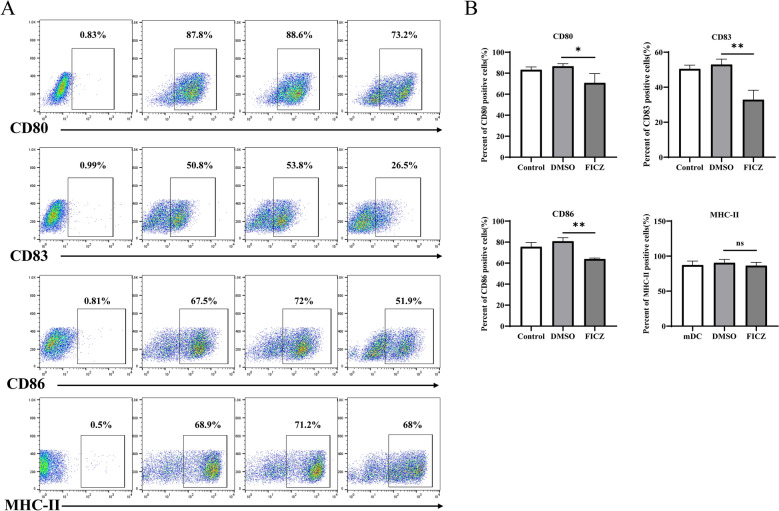
Flow cytometric analysis of the maturation status of FICZ-treated DCs. After the different treatments, the DCs were labeled with fluorescein-conjugated anti-CD80, CD83, CD86 antibodies and major histocompatibility complex (MHC)-II. Flow cytometry was used to detect the expression levels of surface costimulatory molecules and MHC-II. Data in each histogram are expressed as the Mean ± SD of three independent experiments. **A** Flow cytometry analysis; **B** Relative proportion statistical analysis. ^*^P < 0.05, ^**^P < 0.01 versus the control group. ns, not significant

Moreover, we evaluated the surface markers such as costimulatory molecules CD80, CD83, CD86, and MHC-II of the FICZ-treated DCs using flow cytometry. As shown in Fig. 3A, the control DCs and DMSO-treated DCs expressed high levels of CD80, CD83, and CD86. FICZ significantly decreased the levels of CD80, CD83, and CD86 compared with control and DMSO (Fig. 3A, B ). FICZ had no significant effects on the MHC-II expression in the DCs. These results showed that AhR-activated DCs had low expression of costimulatory molecules.

### AhR activation alters cytokine profiles of dendritic cells

**Fig. 4:**
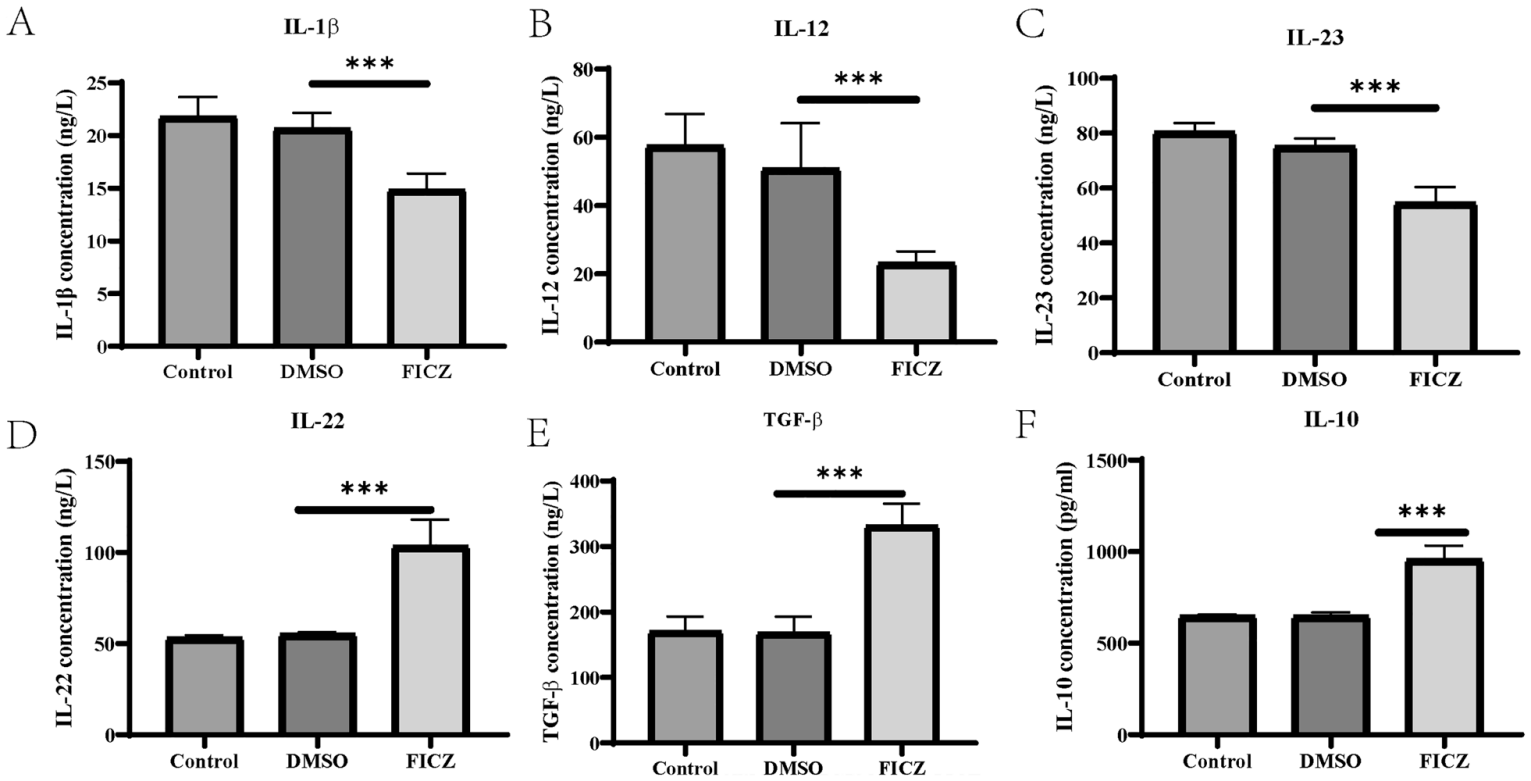
The influence of AhR activation on cytokine production in the supernatant of the treated DCs. The BMDCs were cultured with different cytokines and then treated with 100 nM FICZ for 12 h to activate AhR, followed by stimulation with LPS for another 24 h. The levels of IL-1β, IL-12, IL-23, IL-22, TGF-β, and IL-10 in the supernatants of the different DCs were detected by ELISA. The results are shown as follows: **A** IL-1β; **B** IL-12; **C** IL-23; **D** IL-22; **E **TGF-β; **F** IL-10. Data were collected from three independent experiments. ^***^P < 0.001 versus the control group

**Fig. 5:**
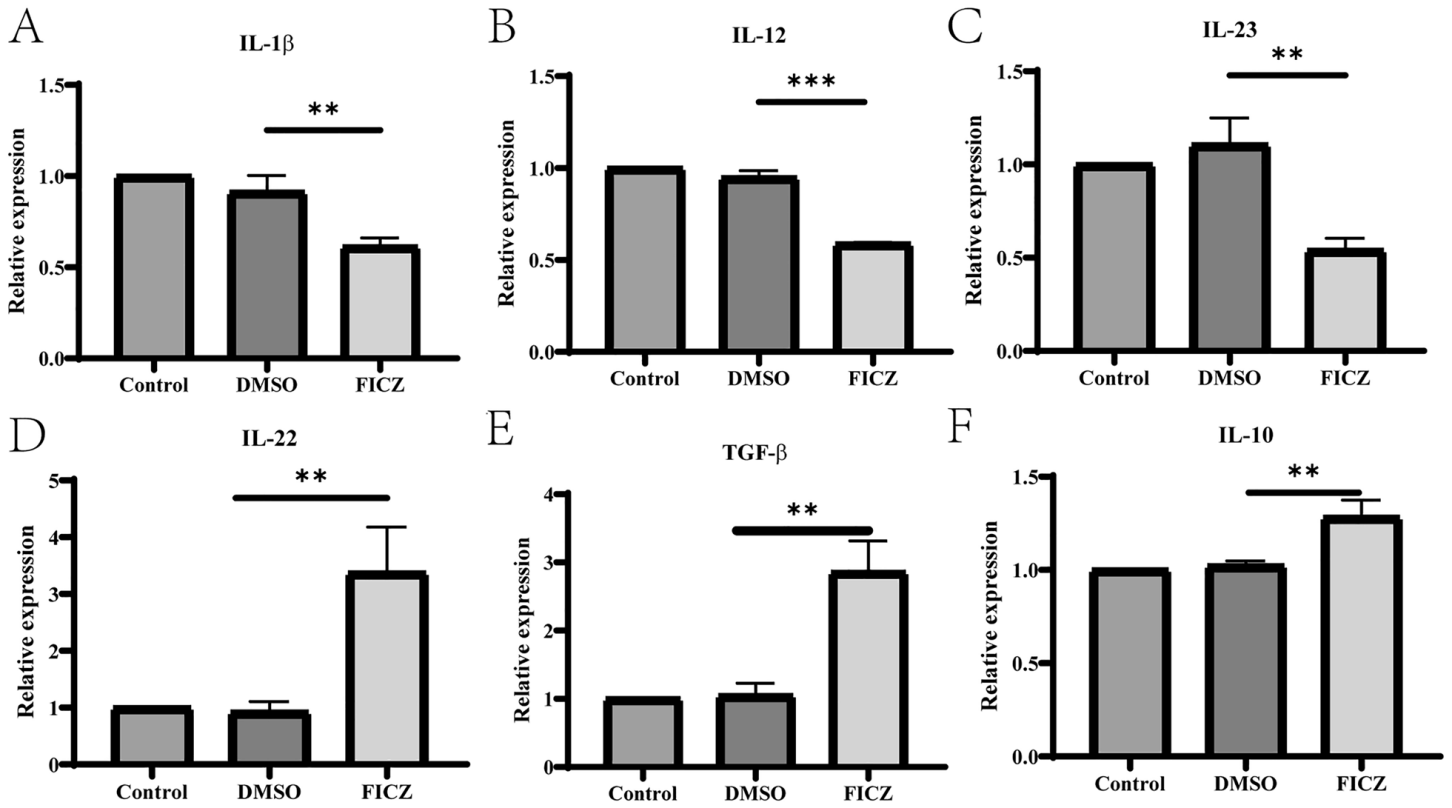
The mRNA levels of cytokines in DCs. The BMDCs were cultured with cytokines and then treated with 100 nM FICZ for 12 h, followed by stimulation with LPS for another 24 h. The mRNA levels of related cytokines were measured using RT-qPCR. GAPDH gene was used as internal control. Data were expressed as Mean ± SD of three independent experiments. The results are shown as follows: **A** IL-1β; **B** IL-12; **C** IL-23; **D** IL-22; **E** TGF-β; **F** IL-10. Data were collected from three independent experiments. ^**^P < 0.01, ^***^P < 0.001 versus the DMSO group. ns, not significant

To confirm whether AhR activation affected the production of cytokines in the DCs, the concentrations of pro-inflammatory (IL-1β, IL-12, and IL-23) and anti-inflammatory cytokines (IL-22, TGF-β, and IL-10) were detected by ELISA. Cytokine analysis indicated that the levels of IL-1β, IL-12, and IL-23 were significantly reduced (Fig. 4A–C). However, the levels of IL-22, TGF-β, and IL-10 secretion were significantly increased (Fig. 4D–F) in the DCs with FICZ-mediated AhR activation. In line with the ELISA results, RT-qPCR results showed that the mRNA levels of IL-1β, IL-12 and IL-23 were lower, while those of IL-22, TGF-β, and IL-10 were higher in the FICZ-treated DCs compared to the DMSO-treated or control DCs (Fig. 5A–F).

### AhR-activated DCs can inhibit CD4^+^ T cell proliferation in vitro

**Fig. 6:**
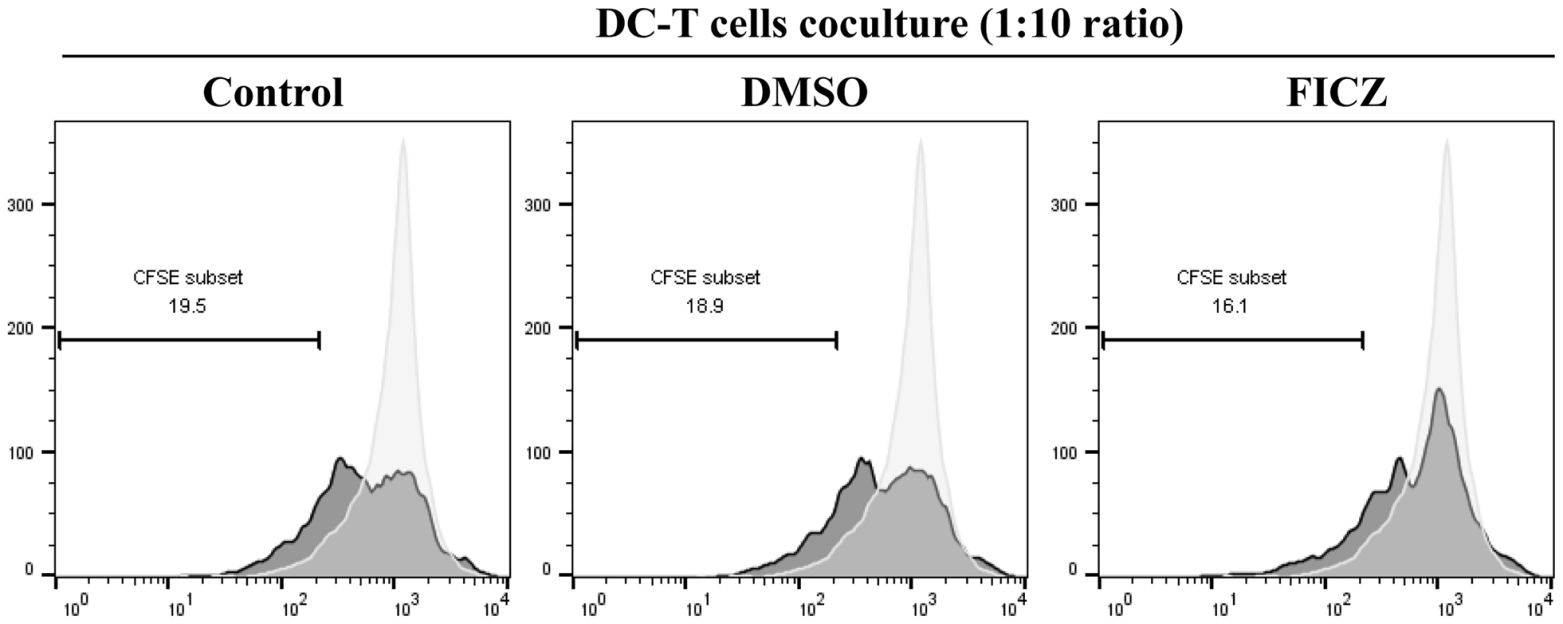
The impairment of AhR activation induces DCs to prime T cell proliferation. The GM-CSF/IL-4-differentiated DCs were treated with or without 100 nM FICZ and then exposed with LPS to induce maturation of DCs for the next 24 h. The conditioned DCs were co-cultivated with autologous CFSE-labeled CD4^+^ T cells for 72 h (1:10 ratio of DC: CD4^+^ T), and T cell proliferation was determined by flow cytometry. The representative plots are shown in the figure

To investigate the immunoregulatory capacity of the AhR-activated DCs on the T cell functions, the DCs conditioned with different factors were cocultured with allogeneic CD4^+^ T cells isolated from the spleen, which were stained with CFSE for three days by adding 2 μg/ml anti-CD3 in coculture medium. Using flow cytometry, T cell proliferation was examined. As shown in Fig. 6, control DCs strongly stimulated the allogeneic T cell proliferation. Slight inhibition of allogeneic antigen-stimulated T cell proliferation was observed when the DCs were treated with FICZ. These data indicate that the AhR-activated DCs may inhibit CD4^+^ T cell proliferation in coculture assays and reduce the number of T cells to further exhibit the tolerance characteristics.

### AhR-activated DCs can promote the differentiation of Treg cells in vitro

**Fig. 7:**
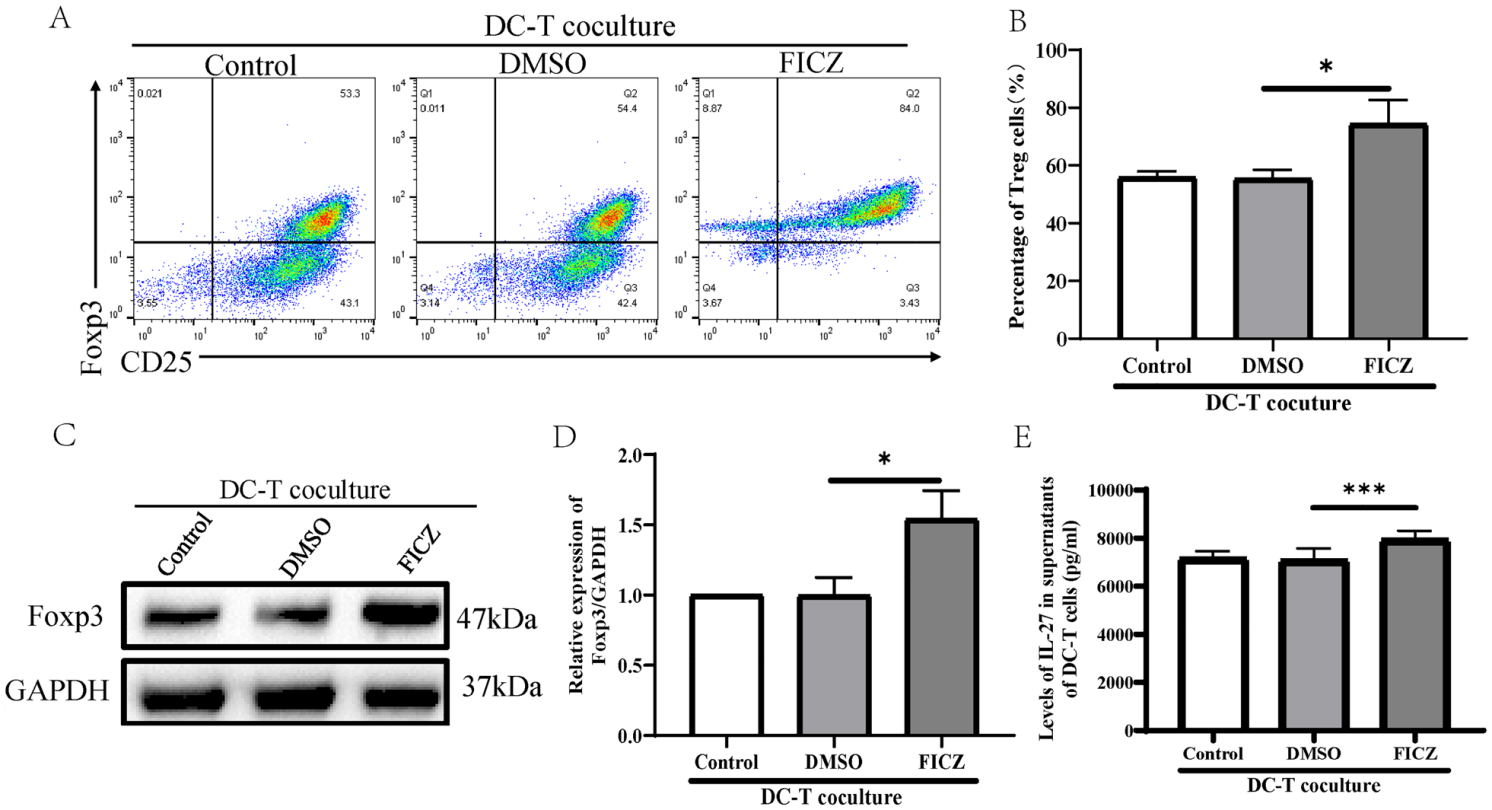
The AhR activated DCs promote Treg cell differentiation. The GM-CSF/IL-4-differentiated DCs were treated with or without 100 nM FICZ and then exposed with LPS to induce the maturation of DCs for the next 24 h. The control, DMSO, and FICZ-treated DCs were cocultured with CD4^+^ T cells for five days by adding IL-2 (100U/ml) and TGF-β1 (5 ng/ml) to the culture medium. The percentage of Treg cells was determined by flow cytometry, the expression of Foxp3 was detected by Western blotting, and the levels of IL-10 in the cocultured supernatant were measured by ELISA. Each experiment was repeated three and the data were collected from independent experiments for analysis. **A** Representative plots of Treg cells shown by flow cytometry; **B** Summarized data for the percentage of Treg cells presented and analyzed as Mean ± SD; **C** Image plot of Western blotting for Foxp3 expression. GAPDH was used as an internal control; **D** The relative intensity for bands of Foxp3 was normalized to GAPDH; **E** ELISA analysis of the levels of IL-10 in the supernatant of cocultured DC-T cells. ^*^P < 0.05, ^***^P < 0.001 versus the DMSO group. ns, not significant

AhR activation has been proven to prime naïve T cells to differentiate into Treg cells and inhibit Th17 cell differentiation, and further modulate the balance between the Treg and Th17 cells. However, it is still controversial whether the AhR-activated DCs influence T cell polarization. Therefore, to clarify the effects of DCs on T cell differentiation, the conditioned DCs were co-cultured with CD4^+^ T cells to determine whether the FICZ-treated DCs could influence Treg cell differentiation and related cytokine secretion. Flow cytometric analyses showed an increased frequency of Treg cells in the FICZ-treated DCs compared with control and DMSO-treated DCs (Fig. 7A, B). Additionally, the expression of Foxp3 protein was upregulated in the CD4^+^ T cells co-cultured with FICZ-treated DCs compared with control and DMSO-treated DCs (Fig. 7C, D). Further analysis revealed that the secretion levels of IL-10 in the supernatants of T cells co-cultured with FICZ-treated DCs was higher than that in the T cells cocultured with control and DMSO-treated DCs (Fig. 7E). These findings suggest that AhR activation may promote the regulation of Treg cell differentiation by DCs.

### Adoptive transfer of tolDCs alleviates the severity of TNBS-induced colitis

**Fig. 8:**
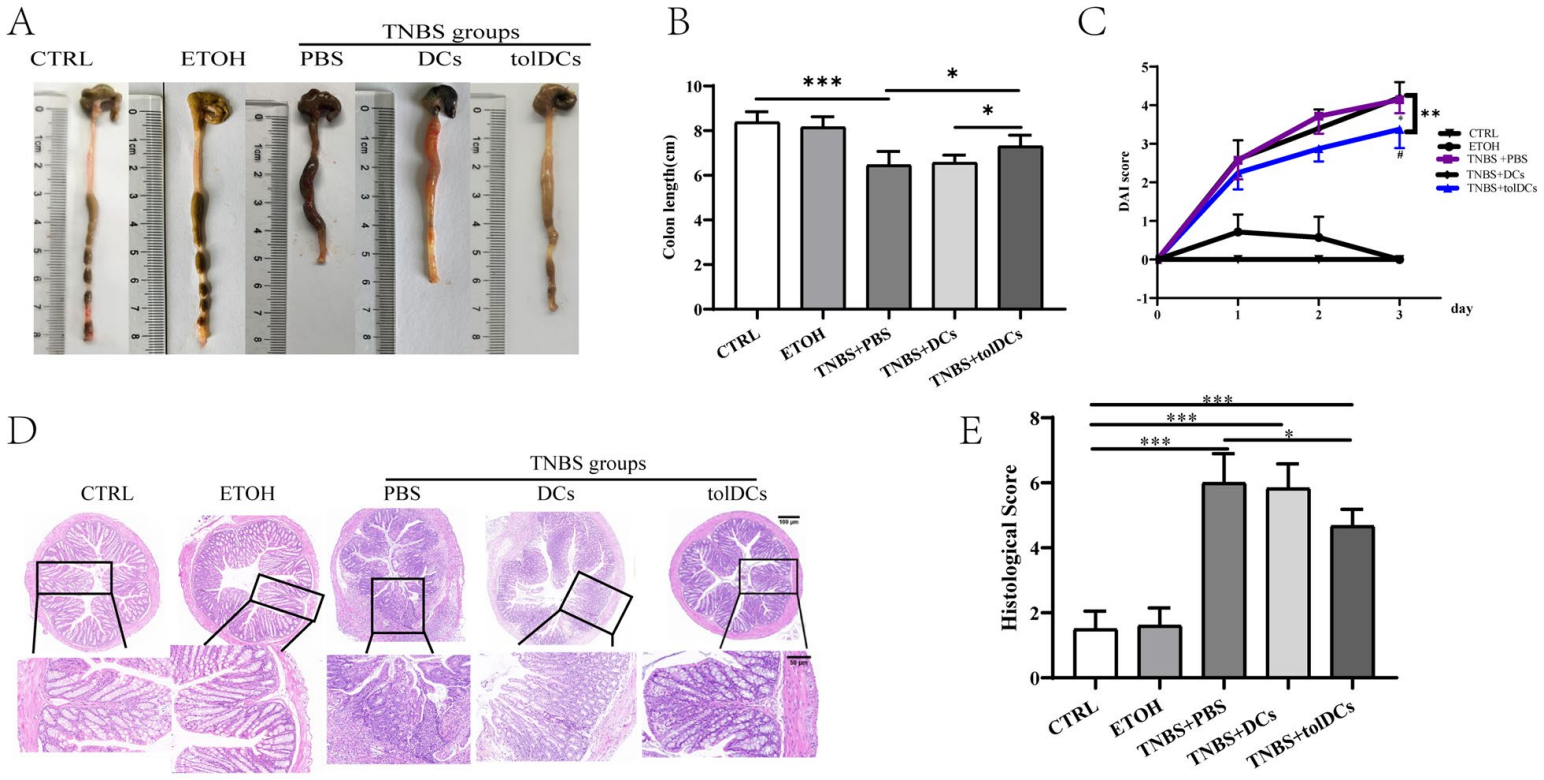
Treatment with tolDCs ameliorates TNBS-induced acute colitis in mice. Experimental colitis was induced with TNBS. The tolDCs were generated in the presence or absence of FICZ as described previously and administered intraperitoneally (i.p.) to the colitis mice 2 h after administration of TNBS. The control mice were injected an equal volume of sterile PBS intraperitoneally. **A**, **B** The general appearance and length of the colon; **C** Data analysis of disease activity index (DAI) for experimental colitis mice on day 3. **D**, **E** Image of histological evaluation, collection and analysis of histological scores, scale bar = 100 μm (above row) and scale bar = 50 µm (below row). All values are expressed as Mean ± SD. n = 5–8 mice per group. ^*^P < 0.05, ^**^P < 0.01, ^***^P < 0.001

The above results indicate that the DCs with AhR activation by FICZ may be considered as tolDCs. TolDCs exhibited tolerogenic properties in vitro. To further confirm the functions of tolDCs in vivo, we assessed whether the tolDCs played a vital role in controlling TNBS-induced colitis after disease onset. TolDCs were generated in the presence of FICZ as described above and then injected intraperitoneally (i.p.) into colitis mice 2 h after administration of TNBS. As shown in Fig. 8, compared with PBS injection, a single injection of 1 × 10^6^ tolDCs relieved the shortening of colon length (Fig. 8A, B). Meanwhile, the adoptive transfer of tolDCs significantly decreased the DAI scores (Fig. 8C) and histology scores (Fig. 8D–E). These results indicate that tolDCs induced by the activation of AhR in vitro effectively relieve colitis, however, the underlying mechanisms are still unclear.

### Transfer of tolDCs results in the restoration of balance between Treg and Th17 cells in TNBS-induced colitis

**Fig. 9:**
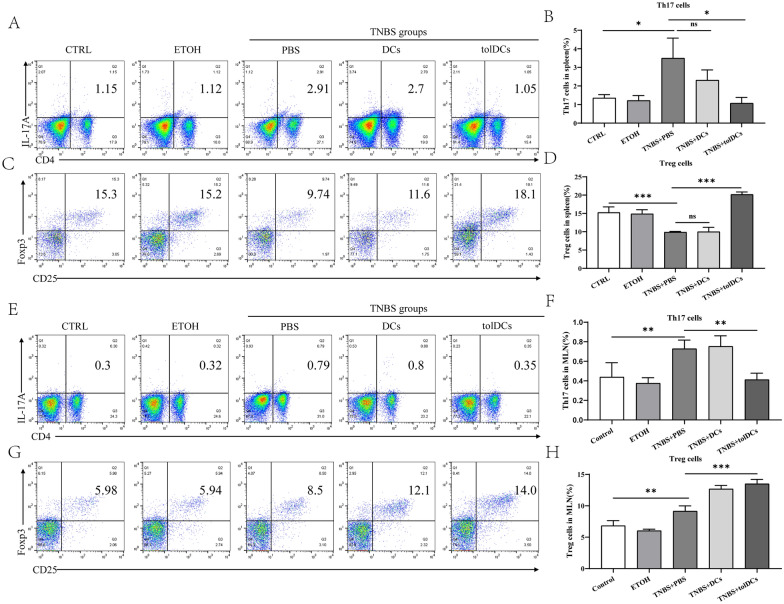
Adoptive transfer of tolDCs restores the balance of Th17/Treg cells in experimental colitis mice. On day 3 after TNBS administration, the mice from each group were euthanized to collect cells from the spleen, and MLNs were collected and stained with anti-CD4, anti-CD25, intracellularly stained with anti-IL-17A and anti-Foxp3. The percentages of Th17 and Treg cells were measured by flow cytometry. Data are shown in the form of flow cytometry chart or expressed as the Mean ± SD (n = 5 mice per group). **A**, **C** The representative plots of Th17 and Treg cells in spleen. **B**, **D** Quantitative analysis of the percentages of Th17 and Treg cells in spleen. **G** The representative plots of Th17 and Treg cells in MLNs. **F**, **H** Quantitative analysis of the percentages of Th17 and Treg cells in MLNs. MLNs, mesenteric lymph nodes. ^*^P < 0.05, ^**^P < 0.01, ^***^P < 0.001. ns, not significant

Adoptive transfer of tolDCs reduced intestinal inflammation in murine colitis, however, the underlying mechanisms are still unclear. Therefore, we examined the Th17/Treg cell balance in colitis mice injected with tolDCs. After injection with tolDCs or PBS as vehicle control, the frequency of Th17 and Treg cells in the spleen and MLNs was determined by flow cytometry. As shown in Fig. 9A, B, an increase in Th17 cells and a decrease in Treg cells were observed in the spleens of colitis mice. The population of Th17 cells in colitis mice injected with tolDCs was decreased compared with PBS-treated colitis mice. The colitis mice injected with tolDCs exhibited an increase in the population of Treg cells (Fig. 9C, D). To determine whether tolDCs restore the imbalance of Th17/Treg cells in MLNs, we detected the proportion of Th17 and Treg cells in MLNs by using flow cytometry. The results indicated that the proportion of Th17 cells in the MLNs of colitis mice was increased. After colitis mice were adoptively transferred with tolDCs, the proportion of Th17 cells in MLNs decreased. Interestingly, the proportion of Treg cells in the MLNs of colitis mice was higher than that in the control group, which showed no obvious intestinal inflammation. The increased proportion of Treg cells in the MLNs of mice with colitis might be a compensatory response to control severe injury. However, cells isolated from MLNs in colitis mice injected with tolDCs were detected the level of Th17 and Treg cells, and the percentage of Treg cells was significantly increased compared with PBS-treated colitis mice (Fig. 9E–H). These data suggest that tolDC injection in colitis mice may alleviate intestinal inflammation by restoring the differentiation balance of Th17/Treg cells.

## Discussion

Accumulating evidence indicates that the function of DCs is dependent on their state of maturation, and that the function of mature DCs is immunogenic, whereas immature or semi-mature DCs are less immunogenic or may even exhibit tolerogenic capability [18, 19]. Therefore, immunogenic DCs play a crucial role in the priming and polarization of CD4^+^ T cells into effector T helper (Th), which participate in regulating immune responses [20, 21]. TolDCs drive T cells to differentiate into Treg cells and regulate peripheral tolerance [21, 22]. CD28 is expressed in T cells and can bind with costimulatory molecules in DCs to trigger T cell activation [23]. TolDCs show low expression of co-stimulatory molecules such as CD80 and CD86. Due to their lack of sufficient co-stimulatory molecular signaling pathways mediated by CD80 and CD86, this kind of DCs triggered effector T cells to be disabled or even apoptosis, prime T cells differentiate into Tregs, and then play an important role in immune suppression [24]. The differentiation of Treg cells relies on the DC-secret IL-10 and TGF-β. To date, numerous drugs and factors have been used to induce and maintain DCs tolerogenic by enhancing their capacity to promote activation of Treg cells [25, 26]. Strategies for repairing T cell immune homeostasis are expected to be translated into clinical application in the treatment of inflammatory bowel disease (IBD). Previous studies have demonstrated that AhR activation induces functional Tregs, which suppress the development of experimental transplant and autoimmune diseases [27, 28]. However, others have reported that FICZ directly promotes Th17 cell production by AhR activation in vitro [12]. In this study, the DCs were found to have reduced proinflammatory cytokines and increased anti-inflammation cytokines and subsequently enhanced the differentiation of Treg cells.

In accordance with previous studies, our results indicated that DCs treated with endogenous ligands FICZ exhibited decreased dendrite and downregulated expression of costimulatory molecules such as CD80, CD86, and CD83, inhibited pro-inflammatory cytokines such as IL-β, IL-12, and IL-23, which might further contribute to Th17 cell differentiation. The levels of IL-10 in the supernatants of FICZ-treated DCs increased. In turn, AhR-activated DCs converted T cells to Treg cells under co-culture conditions in a DC-T cell co-culture system. However, when co-cultured with AhR-activated DCs, the proliferation of CD4^+^ T cells was only slightly suppressed (from 18.9 to 16.1%), which may not be the main mechanism of tolerance induction. Furthermore, we found that the potent anti-inflammation of AhR activation may be involved in the maturation and function of DCs. These data are similar to those by Kayama [29] and Aoki [30] which reported that AhR activation could regulate the maturation and function of DCs an induce tolDCs by promoting Treg cells resolution and function.

The role of DCs in initiating autoimmune diseases and transplant rejection, especially in IBD, which characterize intestinal inflammation. Many efforts have been made to investigate the possible attractive avenue for clinical applications of tolDCs by using animal models of experimental colitis. In this study, we used FICZ to increase the activity of AhR in DCs and produce tolerogenic DCs. Previous studies have investigated the transfer of other kinds of tolDCs induced by immunomodulators to colitis mice significantly reduced the severity of colon inflammation [30–32]. In humans, ex vivo tolDC injection has been applied to clinical type 1 diabetes [33] and multiple sclerosis [34]. The clinical trial in patients with CD is on the way. In this study, to determine the tolerogenic DCs induced by FICZ treatment on the suppression of inflammation in TNBS-induced colitis, we performed adoptive transfer of the tolDCs to colitis mice. The results showed that the tolDCs induced by the AhR ligand FICZ prevented colon inflammation by regulating the Th17/Treg balance in colitis models.

Our data highlight the importance of crosstalk between tolerogenic DCs and T cells in colitis. This study has some limitations. First, in this study, DCs were cocultured with T cells to detect their differentiation, but DCs and T cells were not cultured in separate chambers to further verify the importance of costimulatory molecules. In addition, intestinal cytokine secretion in mice was not detected after DC injection. Finally, the levels of infiltrating Th17 cells and Treg cells in suffering colons were not detected. Therefore, it cannot be directly proved that adoptive transfer of tolDCs regulates the balance of Th17 and Treg in the colon. In the future, we will further confirm the molecular mechanisms related to the induction and function maintenance of DCs by AhR activation and provide a theoretical basis for its potential clinical application.

## Conclusion

In vitro, BMDCs were treated with a kind of AhR ligand, FICZ, and the treated DCs were differentiated to tolerogenic properties by characterization of low expression of costimulatory molecules, low secretion of pro-inflammatory cytokines, and high anti-inflammatory cytokines. Furthermore, the induced tolerogenic DCs effectively enhanced Treg cell differentiation, and the adoptive transfer of tolDCs inhibited immune responses by balancing Th17/Treg cells in a mouse model of colitis. Therefore, our findings may provide new targets for the induction of immune tolerance and control of intestinal inflammation in patients with CD. In conclusion, the possibility of generating tolerogenic DCs by AhR ligand opens a new therapeutic avenue for the treatment of CD.

## Materials and methods

### Mice

Male specific pathogen-free (SPF) C57BL/6 mice (6–8 weeks old, 18–20 g weight, n = 10/each group) were purchased from the Laboratory Center of Nanjing University (Jiangsu, China). All the mice were kept in the Nanjing Medical University Animal Care Facilities and were raised in clean cages under SPF environment with a 12 h light–dark cycle. The mice were given one-week period of adaptation prior to the experiment. All experimental procedures were approved by the Experimental Animal Ethics Committee of Nanjing Medical University (Ethics number: IACUC-1901005).

### Culturing of DCs derived from bone marrow progenitors

As previously described [35], bone marrow DCs (BMDCs) were generated from the murine bone marrow (BM). Briefly, the BM cells were flushed with RPMI1640 medium (Gibco) and passed through a 40 μm nylon mesh to remove pieces of bone and debris as well as the erythrocytes. Further, the BM cells were cultured in RPMI 1640 medium containing 1% penicillin–streptomycin (NCM Biotech, C125C8) supplemented with 10% fetal bovine serum (FBS, Biological Industries, 04-001-0A), 20 ng/ml granulocyte–macrophage colony-stimulating factor (GM-CSF, 315–03), and 10 ng/ml murine recombinant c (IL-4, Peprotech, 214–14). The medium containing fresh cytokines was added to the cells every three days. On Day-6, the BMDCs were purified using magnetic anti-CD11c beads (Stem cell, 18780A). The purified DCs were treated with 100 nM FICZ or dimethyl sulfoxide (DMSO) (< 0.1%) as vehicle control for 12 h and then activated with 100 ng/ml lipopolysaccharide (LPS, Sigma-Aldrich) for another 24 h.

### Scanning electron microscopy

The treated (with DMSO or FICZ) and untreated (control) DCs were fixed in 2.5% glutaraldehyde, dehydrated in ethanol, and critical point dried including fixation and dehydration, transferring from pure acetone to the intermediate solution isoamyl acetate, then replacing isoamyl acetate with liquid carbon dioxide for the samples. The samples were then mounted on aluminum stubs and coated with gold by a sputtering device (Emitech, Ashford, GB). The final cell specimens were observed using a JSM 6700F scanning electron microscope (Jeol, Peabody, MA) at 2.5 kV.

### T cell proliferation experiment

The CD4^+^ T cells were isolated from the spleen of male C57BL/6 mice (6–8 weeks, 18–20 g) by using a T cell isolation kit (BD Biosciences, Catalog: 551539) and labeled with carboxyfluorescein diacetate succinimidyl ester (CFSE, 2.5 μM; Invitrogen, Catalog: C34570). The labeled CD4^+^ T cells were washed twice with RPMI 1640 complete medium and subsequently co-cultured with The treated (with DMSO or FICZ) and untreated (control) DCs. DCs and CD4^+^ T cells were co-cultured at a 1:10 ratio for 72 h (stimulator cells: responder cells = 1:10) by adding 2 μg/ml anti-CD3. The proliferation of CD4^+^ T cells was then analyzed by flow cytometry.

### Flow cytometry

The DCs were stained with fluorescent-labeled antibodies against CD11c, CD80, CD83, CD86, MHC-II, or isotype-matched controls (BioLegend, San Diego, CA, USA). Briefly, the cells were incubated with the antibodies for 30 min at 4 ˚C and subsequently washed with PBS containing 1% FBS. The mature markers CD11c, CD80, CD83, CD86, MHC-II of DCs were analyzed. For Th17 cell analysis, the cells were stimulated with phorbol 12-myristate 13-acetate (PMA, 50 ng/mL, Sigma, P8139) and ionomycin (1 μM, Sigma, Catalog: I3909) in the presence of GolgiPlug (BD, 555029) for 4 h to detect IL-17A. For intracellular staining, the cell surface staining was performed by incubating the cells with FITC-conjugated anti-CD4 (Invitrogen, 11-0042-82) and APC-conjugated anti-CD25 (Invitrogen, 17-0251-81). Further, the cells were fixed, permeabilized, and stained with PE-conjugated anti-Foxp3 (Invitrogen, 12-5773-82) or PE-conjugated anti-IL-17A (Invitrogen, 12-7177-81) using a Transcription Factor Fixation/Permeabilization Kit (eBioscience, 00-5123). All data were analyzed using FlowJo software (Treestar, Ashland, OR, USA) [36].

### T cell differentiation

CD4^+^ T cells were purified from the spleens and lymph nodes of male C57BL/c mice using a CD4^+^ T cell isolation kit (BD Biosciences, 551539) by positive selection according to the manufacturer’s instructions. The cells were then stimulated with 2 μg/mL anti-CD3 (eBioscience, 16-0031-85) and cocultured with 2 × 10^4^ DCs for five days. To induce Treg polarization, IL-2 (100 U/ml) and TGF-β1 (5 ng/ml) were added to the culture medium [37], and the cells cultured under the polarization conditions were harvested and analyzed by flow cytometry.

### Enzyme-linked immunosorbent assay (ELISA)

Cytokines (IL-1β, IL-12, IL-23, IL-22, TGF-β, and IL-10) in culture medium were determined by enzyme-linked immunosorbent assay (ELISA) using specific kits according according to the manufacturer's instructions. All the ELISA kits were purchase from Neobioscience Technology Co., Ltd., China.

### Western blotting

The cells were washed twice with PBS and then lysed in RIPA Lysis Buffer (Beyotime, China) supplemented with a protease inhibitor cocktail (Medchem Express, Nanjing, China). The protein concentrations were determined using an Enhanced BCA Protein Assay Kit (Beyotime, China). Quantitative protein samples (30 μg/lane) were separated on 10% sodium dodecyl sulfate–polyacrylamide gels and transferred to PVDF membranes. After blocking with 5% nonfat milk, the membranes were incubated overnight with anti-CYP1A1 antibody (Proteintech Group, Inc., 1:1000, 13241-1-AP), anti-FOXP3 antibody (Abcam, 1:1000, ab54501), and anti-GAPDH antibody (Bioworld Technology, Inc., 1:10000, AP0063) at 4˚C. Following incubation with horseradish peroxidase-conjugated goat anti‑rabbit antibodies (Bioworld Technology, Inc., 1:10000, BS13278), protein blotting was visualized using an Enhanced Chemiluminescence (Life Technologies, Carlsbad, USA). Data ware analyzed using Image-Lab 6.0.1 software (Bio-Rad).

### Real-time quantitative polymerase chain reaction (RT-qPCR)

RNA was isolated using TRIzol^®^ reagent (Life Technology) and purified using a gDNA wiper kit. cDNA was synthesized by reverse transcription from 1 µg RNA using a HiScript^®^ II Q RT SuperMix, and RT-qPCR was performed on an ABI 7300 Real-Time PCR System (Thermo Scientific) via ChamQ Universal SYBR qPCR Master Mix. All reagents were obtained from Vazyme Biotech Co., Ltd. The primers for the related genes were designed and synthesized by Qingke Biotechnology Co., Ltd. In the present study, the following primers were used (mouse): IL-1β forward 5-AAGGGGACATTAGGCAGCAC-3 and reverse 5-ATGAAAGACCTCAGTGCGGG-3; IL-12 forward 5- GGGACCAGGCCCTATTATGC -3 and reverse 5- TTGCATCCATTTGTGTGGCG -3; IL-23 forward 5-AAATAATGTGCCCCGTATCCAG-3 and reverse 5-GAAGATGTCAGAGTCAAGCAGGTG-3; IL-22 forward 5-ATGAGTTTTTCCCTTATGGGGAC-3 and reverse 5- GCTGGAAGTTGGACACCTCAA-3; TGF-β forward 5- CAATTCCTGGCGTTACCTTG-3 and reverse 5-AGCCCTGTATTCCGTCTCCT-3; IL-10 forward 5-GCTCTTACTGACTGGCATGAG-3′ and reverse 5-CGCAGCTCTAGGAGCATGTG-3; GAPDH forward: 5-AGGTCGGTGTGAACGGATTTG-3 and reverse 5-TGTAGACCATGTAGTTGAGGTCA-3′. The relative gene expression was determined using 2^−△△CT^, and the housekeeping gene GAPDH was used as a reference control.

### Induction of experimental colitis by TNBS

Experimental colitis was induced using TNBS according to a previously described protocol [38, 39]. Briefly, C57BL/c mice were fasted, with free access to 5% glucose water, for 24 h before administering the enema, and were then randomly assigned to five groups (n = 10/per group). Mice were lightly anesthetized by inhalation of low-dose ether. TNBS was diluted to 50% with an equal volume of 100% ethanol. In the TNBS group, each mouse was treated with 100 μl 50% TNBS by slighting inserting a catheter to colon about 4 cm from the anus and was maintained to a head-down position for about 1 min to prevent drug solution from flowing out. In the vehicle-treated group, each mouse was infused with an equal volume of 50% ethanol. The disease activity index (DAI) was used to evaluate colonic damage including weight loss, stool consistency, and bloody stool, as described previously [40]. Histological scores of colon HE staining were calculated using a method described in previous studies [41, 42].

### Adoptive transfer experiment

The BMDCs were pretreated with 100 nM FICZ and then subsequently activated by LPS to induce tolDCs in vitro as mentioned above. Then, the tolDCs were collected and washed three times with sterile PBS to remove any remaining FICZ. To assess the effect and mechanism of tolDCs on colitis, 1 × 10^6^ tolDCs or control DCs were injected intraperitoneally to colitis mice. The vehicle mouse was injected intraperitoneally with an equal volume of sterile PBS.

### Statistical analysis

All data are expressed as mean ± standard deviation (SD) and analyzed using SPSS 25.0 software (IBM Company, Armonk, NY). The differences among groups were analyzed using one-way analysis of variance (ANOVA) and Bonferroni correction. All graphs were drawn using Graphpad software 8.0 (GraphPad Inc., San Diego, CA). A *P*-value lower than 0.05 was considered statistically significant.

## Data Availability

The original data supporting this research conclusion will be availability from the corresponding author.

## Abbreviations

IBD: Inflammatory bowel disease
CD: Crohn’s disease
DCs: Dendritic cells
AhR: Aryl hydrocarbon receptor
tolDCs: Tolerogenic DCs
DAI: Disease activity index
Th17: T helper 17 cells
Treg: Regulatory T cells
MHC: Major histocompatibility complex
NF-κB: Nuclear factor κB
ITE: 2- (1'H-indole-3'-carbonyl)-thiazole-4-carboxylic acid methyl ester
3-MC: 3-Methylcholanthrene
FICZ: 6-Formylindolo [3,2-b] carbazole
TCDD: 2,3,7,8-Tetrachlorodibenzo-p-dioxin
ARNT: Aryl hydrocarbon receptor nuclear translocator
TNBS: 2,4,6-Trinitrobenzenesulfonic acid H&E hematoxylin–eosin
BMDCs: Bone marrow derived dendritic cells FBS fetal bovine serum
DMSO: Dimethyl sulfoxide
ELISA: Enzyme-linked immunosorbent assay RT-qPCR real time quantitative PCR
GM-CSF: Granulocyte–macrophage colony-stimulating factor IL-4 interleukin (IL)-4
CFSE: Carboxyfluorescein diacetate succinimidyl ester
PMA: Phorbol 12-myristate 13-acetate
SD: Standard deviation
MLNs: Mesenteric lymph nodes
